# Fetal–Maternal Exposure to Endocrine Disruptors: Correlation with Diet Intake and Pregnancy Outcomes

**DOI:** 10.3390/nu12061744

**Published:** 2020-06-11

**Authors:** Alessandro Rolfo, Anna Maria Nuzzo, Ramona De Amicis, Laura Moretti, Simona Bertoli, Alessandro Leone

**Affiliations:** 1 Department of Surgical Sciences, University of Turin, Via Ventimiglia 3, 10126 Turin, Italy; a.nuzzo@unito.it (A.M.N.); l.moretti@unito.it (L.M.); 2 International Center for the Assessment of Nutritional Status (ICANS), Department of Food, Environmental and Nutritional Sciences (DeFENS), University of Milan, Via Sandro Botticelli 21, 20133 Milan, Italy; ramona.deamicis@unimi.it (R.D.A.); simona.bertoli@unimi.it (S.B.); alessandro.leone1@unimi.it (A.L.); 3 Istituto Auxologico Italiano, IRCCS, Lab of Nutrition and Obesity Research, 20145 Milan, Italy

**Keywords:** endocrine-disrupting chemicals (EDCs), bisphenol A, phthalats, pregnancy, placenta, diet, pregnancy pathologies

## Abstract

Endocrine-disrupting chemicals (EDCs) are exogenous substances able to mimic or to interfere with the endocrine system, thus altering key biological processes such as organ development, reproduction, immunity, metabolism and behavior. High concentrations of EDCs are found in several everyday products including plastic bottles and food containers and they could be easily absorbed by dietary intake. In recent years, considerable interest has been raised regarding the biological effects of EDCs, particularly Bisphenol A (BPA) and phthalates, on human pregnancy and fetal development. Several evidence obtained on in vitro and animal models as well as by epidemiologic and population studies strongly indicated that endocrine disruptors could negatively impact fetal and placental health by interfering with the embryonic developing epigenome, thus establishing disease paths into adulthood. Moreover, EDCs could cause and/or contribute to the onset of severe gestational conditions as Preeclampsia (PE), Fetal Growth Restriction (FGR) and gestational diabetes in pregnancy, as well as obesity, diabetes and cardiovascular complications in reproductive age. Therefore, despite contrasting data being present in the literature, endocrine disruptors must be considered as a therapeutic target. Future actions aimed at reducing or eliminating EDC exposure during the perinatal period are mandatory to guarantee pregnancy success and preserve fetal and adult health.

## 1. Introduction

Natural and man-made chemicals may mimic or interfere with the endocrine system, a complex communication network among the nervous system and key biological functions such as reproduction, immunity, metabolism and behavior [1]. These compounds—known as endocrine-disrupting chemicals (EDCs)—are found in pesticides, metals and in several everyday products, including plastic bottles and food containers, detergents, flame retardants, toys and cosmetics [2]. Due to their extreme diffusion in everyday life, EDCs became object of intense investigation by the medical-scientific community, mainly to clarify their role as risk factors and/or pathogenic triggers. Pregnancy is the most sensitive clinical environment, where two lives, the mother and the developing embryo, could be simultaneously affected by EDC activity. There are contrasting data in the literature about the immediate and long-term effects of maternal and fetal EDC exposure.

Therefore, the objective of this paper is to review the most recent findings on fetal–maternal exposure to endocrine disruptors, particularly focusing on Bisphenol A (BPA) and phthalates, pregnancy outcomes, the in-utero setting of post-natal and adult conditions as well as up-to-date guidelines to reduce dietary EDCs exposure during the perinatal period.

## 2. Endocrine Disrupting Chemicals (EDCs)

According to the World Health Organization (WHO), an endocrine disruptor is “an exogenous substance or mixture that alters function(s) of the endocrine system and consequently causes adverse health effects in an intact organism, or its progeny, or (sub) populations” [3].

EDCs disrupt endocrine functions mainly by mimicking natural hormones like estrogens, androgens or thyroid hormones. Moreover, they could alter hormones’ metabolism, thus blocking and antagonizing their interaction with the specific membrane and/or intra-cellular receptors [2]. Almost 800 chemicals are suspected to interfere with endocrine functions [4]. For example, dioxins are a byproduct in herbicide production, paper bleaching or in waste burning, while perchlorate is a secondary product of aerospace, weapon and pharmaceutical industries frequently found in drinking water [5,6]. Other EDCs are perfluoroalkyl and polyfluoroalkyl (PFAS), used in industrial applications as firefighting foams and non-stick pans [7]. Phytoestrogens are natural endocrine disruptors occurring in plants that have hormone-like activity, such as genistein and daidzein, that are in soy products like tofu or soy milk [8]. Of particular interest are BPA and phthalates, widely investigated as “obesogenic” factors that could interfere with pregnancy physiology and fetal development, thus being a leading cause of reproductive disorders [4]. Fresh meat, fish and vegetables which are not packed in plastic have a low concentration of BPA and phthalates [9], while ready-to-eat and fast food products stored in plastic bags and cans are a major exposure source to these EDCs [10,11].

BPA is an industrial chemical obtained from condensation between phenol and acetone that has been used to produce plastics and resins since the 1960s. It is considered the first synthetic estrogen produced, although it does not possess a steroid structure since it does not include the phenanthrene nucleus [12]. BPA is found in polycarbonate plastics that are commonly used in containers to store food, beverages and other goods. It is also present in epoxy resins used to coat metal products as food cans, bottle tops and water supply lines. Indeed, it is very easy to be exposed to BPA, and since 2011 the European Union prohibited BPA use at least in polycarbonate baby bottles. BPA was recently removed from many consumer products, being replaced by its structural analogs Bisphenol-S (BPS) and Bisphenol-F (BPF). Nevertheless, BPS and BPF have detrimental effects similar to BPA, such as cytotoxicity, genotoxicity, reproductive toxicity, dioxin-like effects and neurotoxicity. Moreover, it has been shown that BPS and BPF exhibit estrogenic and/or anti-androgenic activities similar to or even greater than those of BPA [13,14]. While studies investigating BPS and BPF metabolism are lacking, BPA is absorbed by the digestive tract and, to a lesser extent, by the skin through direct contact [15]. After ingestion, BPA is metabolized by the intestinal microbiota and by the liver, mainly by glucuronidation and, to a lesser extent, sulfation, resulting in the production of BPA monoglucuronide (BPA-G) and BPA sulphate (BPA-S), with BPA-G being the main BPA metabolite in humans [16]. The majority of BPA is metabolized to BPA-G via the Uridine 5′-diphospho-glucuronosyltransferase (UGT) system. Studies on the fetal expression of UGT enzymes responsible for BPA glucuronidation detected no to low enzyme activity during the prenatal period [17]. Nevertheless, the human fetus is exposed to high BPA concentrations, since BPA-G and BPA-S are deconjugated and thus converted back to BPA in the placenta [18].

BPA is well known for its estrogenic activity exerted through the activation of the Estrogen Receptor (ER), with a stronger affinity for ERβ than for ERα, whereas BPA-G did not show ER-mediated estrogenic activity [19], but it exerted pro-inflammatory effects via the competitive inhibition of PPAR-γ signaling [20].

Phthalates are diesters of phthalic acid classified into high- and low-molecular-weight ones. The first category includes several compounds that are largely adopted to increase plastic flexibility and durability. Among them, the most commonly employed additive is di (2-ethylhexyl) phthalate (DEHP). Low-molecular-weight phthalates, like diethyl phthalate (DEP), are mainly used in personal care products and cosmetics, but they could be also found in pesticides and in food packaging [21]. Exposure to DEHP is reflected by the presence of its metabolites in urine, such as mono (2-ethylhexyl) phthalate (MEHP), mono (2-ethyl-5-hydroxyhexyl) phthalate, mono (2-ethyl-5-carboxypentyl) phthalate and mono(2-ethyl-5-oxohexyl) phthalate, whereas the main urinary metabolite of DEP is mono-ethyl phthalate (MEP). Remarkably, the bioactivity of phthalate metabolites is superior to that of the original substance [16].

BPA and phthalates are therefore widely present in everyday life due to the considerable volume of plastic produced [22]. They could be released at room temperature but heating facilitates their leaking out, resulting in massive food and beverage contamination [23]. For example, in polycarbonate (PC) bottles, high temperatures stimulate BPA migration into water by promoting hydrolysis and/or wall permeability [24], with a temperature that influences BPA migration more than heating time [25]. Table 1 summarizes the most important EDCs known to be disease risk factors.

## 3. Exposure to BPA and Phthalates

Worldwide estimations of ECD daily intake based on urinary exposures revealed different exposure levels across continents, countries, regions, and towns [28].

The globally estimated BPA daily intake in adults is 30.76 ng/kg bw/day, whereas a notably higher daily intake, corresponding to 42.03 ng/kg bw/day, was found for pregnant women [29]. Both exposures are below the tolerable daily intake (TDI) of 50 and 4 μg/kg bw/day recommended by both the U.S. Environmental Protection Agency (US EPA) [30] and the European Food Safety Authority (EFSA) [31], respectively. The highest exposures have been estimated for some European countries, including Italy, while African countries seem to be the least exposed [29]. Over 90% of the overall BPA exposure is thought to be due to diet, while the contribution of the non-dietary sources is less than 5% [32]. However, some authors consider that the non-dietary routes might be also important.

As regards phthalate daily intake, China’s adult population seems to be the most exposed [33], with peaks of 60 μg/kg bw/day of DEHP in some coastal areas [34], a value three times higher than the TDI of 50 μg/kg bw/day established by EFSA and the reference dose suggested by the US EPA of 20 μg/kg bw/day. Lower intake levels, within the safety ranges, were estimated for other regions of China and for other countries [35,36,37,38,39]. In Italy, a daily intake of DEHP of 3.1 μg/kg bw was found in Italian hospitalized adults [40]. Contaminated food is likely to be the largest route of exposure to phthalate in the general population [41].

Although providing interesting data to identify populations at a greater exposure risk and to assist policy makers in making regulations to reduce EDC exposure, one has to be aware of several pitfalls when interpreting exposure to EDC based on urinary concentrations. First, the use of an equal body weight for all populations risks overestimating exposure for some countries and underestimating it for others. Second, BPA has been shown to accumulate in adipose tissue, affecting urinary excretion [42,43,44,45]. Thus, differences in the exposure to these EDC may exist because of the different body composition between ethnic groups, ages and sexes. Third, the accuracy of estimates by region, country, or continent depends on the number of studies available for each of them. While for some countries, such as the USA and China, estimates are derived from dozens of studies investigating the ECDs exposure in the population, for others, such as Italy, the estimate is derived from exposure found from one or, at most, two studies.

All of this should be taken into consideration when estimating and interpreting the daily intake of BPA and phthalates.

## 4. EDCs and Reproductive Health

### 4.1. Exposure to EDCs and Fertility

Human reproduction and pregnancy success depend on both female and male reproductive health and EDCs, as BPA and phthalates could affect fertility of both genders.

The ovaries are responsible for female gametogenesis and endocrine functions during reproductive life. It has been demonstrated that prenatal BPA exposure inhibited germ cell nest breakdown in F1 generation ovaries in mice, decreased the numbers of primordial, primary, preantral and total healthy follicles at post-natal day 21 and decreased estradiol levels in female rats dosed for 1 year [46,47]. Hu and colleagues reported that sexually mature CD-1 mice treated by 5 consecutive BPA concentration gradients (1, 10, 100, 1, and 10 mg/kg) for 28 days initiated the excessive premature activation of primordial ovarian follicles via the PTEN/PI3K/AKT signaling pathway by downregulating PTEN expression in vivo [48]. In a previous prospective cohort study, the association between urinary BPA concentration and ovarian response among women undergoing in vitro fertilization (IVF) was investigated. BPA was detected in the majority of IVF women and its urinary concentrations (ranging from <0.4 to 25.5 mg/L) were inversely associated with number of retrieved oocytes per cycle and peak levels of serum estradiol [49]. Moreover, a recent study on 700 Chinese couples attempting pregnancy revealed that women with the highest urinary BPA concentration (>2.33 ng/mL) had a 30% reduction in fecundability and a 64% increase in the odds of infertility [33].

Phthalates were shown to disrupt female fertility by altering oocytes development and maturation. Data are available from animal models. Adult CD-1 mice orally daily dosed with DEHP (20–750 mg/kg/day) for 10 and 30 days showed a decreased percentage of primordial follicles and an increased percentage of primary follicles, thus reducing reproductive lifespan. The mechanism by which DEHP accelerates primordial follicle recruitment is likely via over-activation of the phosphatidylinositol 3-kinase (PI3K) signaling, a pathway that regulates primordial follicle survival, quiescence and recruitment. DEHP exposure increased ovarian mRNA levels of 3-phosphoinositide-dependent protein kinase-1 (Pdpk1), mammalian target of rapamycin complex 1 (Mtorc1), factors that drive primordial follicle recruitment, and decreased Phosphatase and tensin homolog (Pten) and Tuberous sclerosis 1 (Tsc1) mRNA levels, promoters of primordial follicle quiescence [50]. Prenatal DEHP exposure (0–40 μg/kg/day) significantly reduced percentage of methylated CpG sites in Insulin-like growth factor 2 receptor (Igf2r) and Paternally expressed gene 3 (Peg3) differentially methylated regions (DMRs) in fetal primordial germ cells and in the oocytes of the F1 mice. These DEHP-induced oocytes DNA methylation alterations were inherited by the F2 mice, indicating that DEHP effects on oocyte development are heritable [51]. MEHP, the active toxicant DEHP metabolite, significantly reduced mouse oocyte viability at concentrations of 250 and 500 μM by promoting oxidative stress. Overexpression of Cu–Zn superoxide dismutase (Sod1) and decreased expression of mitochondrial respiratory chain protein (Nd1) were identified as possible molecular mechanism leading to these MEHP-induced alterations in oocyte viability [52].

In males, EDCs exposure was associated with declined semen quality, increased sperm DNA damage, alterations in testis morphology and endocrine function [20,53,54,55]. Mantzouki and colleagues demonstrated that very high concentrations of plasma BPA (>3 ng/mL) were associated with azoospermia in humans [56]. A cross-sectional study performed on 215 healthy young university students (18–23 years old) revealed a significant positive association between urinary BPA concentrations (2.8 (0.16–11.5) ng/mL) and serum LH levels as well as a significant and inverse association with sperm concentration and the total sperm count, thus concluding that BPA exposure may be associated with a reduction in Leydig cell capacity and decreased sperm counts in young men [57]. In accordance, other studies demonstrated that urinary BPA levels (median 1.87 µg/L) were associated with decreased sperm concentration and mobility, reduced semen quality, decreased antioxidant levels, reduced sperm DNA integrity and increased percentage of immature sperm [58,59].

Even phthalates could negatively impact on male fertility. DEHP exposure (750 mg/kg/day) in male CD-1 mice induced lower serum testosterone levels, accompanied by higher serum estradiol and LH levels. Moreover, histological mice evaluations showed that male mice prenatally exposed to DEHP were characterized by increased germ cell apoptosis, and the degeneration of seminiferous tubules, oligozoospermia, asthenozoospermia, and teratozoospermia, leading to premature reproductive senescence [60]. Yuan and colleagues described that DBP exposure (500 mg/kg) significantly decreased sperm count in F1 through F3 generations. Specifically, they found global DNA hypomethylation along with the hypomethylation of follistatin-like 3 (Fstl3) promoter, known modulator of Sertoli cell number and spermatogenesis [61]. Recent evidence suggested that embryonic exposure to DEHP (500 mg/kg body weight/day) could disrupt testicular germ cell organization and spermatogonia stem cell function in a transgenerational manner. Specifically, DEHP treatment of pregnant CD1 outbred mice at Embryonic Day 7 (E7) to E14 lead to disrupted testicular germ cell association, decreased sperm count and motility in F1 to F4 offspring [62].

Therefore, EDCs as BPA and phthalates could have severe detrimental effects on human reproduction, impairing both female and male fertility before pregnancy onset and causing gametes anomalies during fetal development that could be inherited by the following generations.

### 4.2. Exposure to EDCs during the Reproductive Age and Development of Obesity, Diabetes and Cardiometabolic Abnormalities

The reproductive age is a critical phase in the life of a woman, as the development of certain pathological conditions during this phase may influence the outcomes of future pregnancies. The presence of obesity [63], diabetes [64,65,66] and cardiometabolic abnormalities [67,68], during pregnancy has been associated with an increased risk of adverse maternal and fetal outcomes, including gestational hypertension, preeclampsia, gestational diabetes, caesarean section, congenital malformations, shoulder dystocia, perinatal death, preterm birth and large for gestational age at birth. A pregnancy free of such comorbidities is, thus, the best situation to preserve the health of both mother and child.

Nevertheless, the prevalence of obesity, diabetes and metabolic syndrome continuing to increase worldwide [69]. Unhealthy dietary habits and a sedentary lifestyle are considered the big two causes of such an increment, but there is a growing body of evidence suggesting a possible relationship between exposure to EDC and cardiometabolic health [25]. It must be said that almost all epidemiologic evidence on this issue comes from large cross-sectional studies and some cohort studies including women of all ages, whereas the evidence of an association between the exposure to EDC during the reproductive age and the risk of developing obesity, diabetes and cardiometabolic abnormalities is limited to few, generally small, cross-sectional studies [70,71,72,73].

A small cross-sectional study involving 103 women aged 19–50 found that urinary BPA was associated with BMI and, marginally, with waist circumference, after adjustment for energy intake and energy requirement [70]. Similarly, another cross-sectional study, involving 246 healthy premenopausal women with regular menstrual cycles, found that the urinary BPA was modestly associated with BMI, waist circumference and fat mass assessed by DXA, after age-adjustment [71]. A third cross-sectional study including 307 Korean women aged 30–49 years found the urinary BPA concentration was associated with BMI, waist circumference, fasting insulin and HOMA index, but not with fasting glucose, after adjustment for age, smoking status, alcohol status, triglyceride, total and HDL cholesterol levels. Contrarily, urinary mono-(2-ethyl-5-hydroxyhexyl) phthalate (MEHHP), mono-(2-ethyl-5-oxohexyl) phthalate (MEOHP) and mono-n-butyl phthalate (MNBP) concentrations were not associated with any anthropometric and metabolic parameter [72]. Differently, the Shanghai Food Consumption Survey (SHFCS) 2012–2014, including 1222 women, observed increased odds of central obesity in women aged ≤ 45 years and with higher urinary levels of mono-2-ethylhexyl phthalate (MEHP) (OR 3.51, 95% CI 1.34, 7.00), MEOHP (OR 3.78, 95% CI 1.28, 8.72), MEHHP (OR 3.82, 95% CI 1.28, 8.84), and mono-(2-ethyl-5-carboxypentyl)phthalate (MECPP) (OR 4.15, 95% CI 1.54, 8.58) compared to women of the same age but with the lowest exposure [73].

At this stage, it is clear that it is not possible to draw conclusions on the relationship between exposure to EDC during reproductive age and the risk of developing pathological conditions potentially affecting pregnancy outcome. However, given the effect that obesity and metabolic complications can have on the health of both mother and child, it is urgent to conduct epidemiological studies specifically designed for this issue.

## 5. EDCs and Fetal Programming

The Developmental Origins of Health and Disease theory (DOHaD) introduced the concept that maternal diet, environmental insults and lifestyle could significantly impact on fetal–placental, development, thus establishing disease paths into adulthood [74,75]. In this context, endocrine disruptors may play a leading role in fetal programming. It was previously reported that the placenta is not such an effective barrier against EDCs and that pregnant women’s exposure was associated with EDCs’ entrance in the fetal circulation [76,77,78]. Importantly, the developing fetus might be more sensitive to EDCs than the adult [76] explaining why endocrine disruptors could adversely affect fetal development with a particular impact on reproductive and hormonal systems. Epigenetic influences were implicated as mediators of the relationship between EDCs, environmental insults and health status, while BPA and phthalates were indicated as sources of epigenetic disruption [79].

Epigenetics defines heritable phenotype changes that involve alterations in gene expression and not in DNA sequence. Epigenetic mechanisms include DNA methylation, acetylation, genomic imprinting, as well as the expression of microRNAs (miRNAs) and non-coding RNAs. These modifications usually have an inhibitory effect on gene expression since they modulate DNA accessibility to transcription factors and regulatory proteins by altering chromatin structure and/or by the recruitment of histone modifiers. Early fetal development is particularly vulnerable to epigenetic insults since it is characterized by a high DNA synthesis rate and because the complex machinery modulating DNA methylation and chromatin organization is established at this time [80].

Studies investigating EDCs’ impact on fetal–placental epigenetics in early human pregnancy are few due to the difficulties of collecting samples during the first trimester of pregnancy. Therefore, most of the data available came from in-vitro and animal studies.

Data obtained on human fetal liver biopsies revealed that even low BPA doses could significantly influence in-utero epigenetic regulation of xenobiotic metabolizing enzymes (XMEs), pivotal for compounds metabolism and excretion. In particular, they described increased methylation at COMT and SULT2A1 promoters related to higher BPA levels, anomalies that could alter disease susceptibility later in life [81].

A recent cohort study reported the negative association among first-trimester maternal exposure to BPA and phthalates and term cord blood methylation of imprinted (H19, IGF2) and non-imprinted (PPARA, ESR1) genes and LINE-1 repetitive elements [82], epigenetic targets associated with growth, development and metabolism. Of note, a sex-stratified analysis for DNA methylation revealed that these EDC-induced effects were female-specific [82], confirming the epigenetics-related sexually dimorphic effects of EDC exposure during prenatal development previously demonstrated in several animal models [83]. A similar designed study was performed on 296 newborns from the CHAMACOS Mexican–American longitudinal birth cohort. They specifically focused on fetal phthalates metabolites’ exposure during pregnancy, assessed on maternal urines, and their association with imprinted genes DNA methylation quantified on cord blood samples at term. Interestingly, this investigation demonstrated a significant positive association between phthalate metabolites concentration and the methylation of Maternally Expressed 3 (MEG3) gene, known for its role in early growth, tumorigenesis and metabolic processes [84].

Moreover, early life EDC exposure could affect obesity epigenetic programming through endocrine disruptors’ ability to bind nuclear receptors as the Peroxisome Proliferator-Activated Receptor (PPAR)γ, master regulator of adipogenesis modulating the expression of metabolic genes during differentiation [85]. EDC-induced obesogenic effects are also accompanied by altered methylation of PPARγ or PPARγ target genes [85]. Indeed, the relative expression of PPARγ-induced genes during early development determines whether mesenchymal stem cells differentiate into osteocytes or adipocytes, thus influencing body fat accumulation [86].

Another EDC target with important consequences for epigenetic regulation is the Estrogen Receptor (ERα) [76,87]. ERα is a transcription factor whose activation triggers estrogen-responsive elements present on the promoter region of the histone-lysine N-methyltransferase enzyme EZH2, key player in gene silencing. It was recently reported that BPA and other estrogenic EDCs, by activating Estrogen Receptors and ERs coregulators Mixed Lineage Leukemia histone methylases (MLL2 and MLL3) and histone acetyltransferase CBP/P300, increase the expression of EZH2 [88], thus potentially affecting global epigenetic regulation, even during in-utero development.

Even the placenta is directly affected by EDC activity. Studies conducted in mice demonstrated that BPA intrauterine exposure markedly altered the placental methylation of imprinted genes [89] affecting placental loss-of-imprinting and decreasing both global and CpG-specific DNA methylation [90]. Furthermore, it was reported in the HTR8/SVneo human cytotrophoblast cell line that BPA exposure negatively affected cytotrophoblast invasion through the hypermethylation and downregulation of the WNT2 gene via DNA (cytosine-5)-methyltransferase 1 (DNMT1), with a negative correlation confirmed also in BPA-induced preeclamptic-like mouse placentae [91]. The BPA-substitute BPS was reported to alter the placental expression of the efflux transporter P-glycoprotein (P-gp), one of the main regulators of fetal exposure to xenobiotics encoded by the ABCB1 gene. Using CRL-1584 human trophoblast cell lines, Speidel and colleagues demonstrated that acute exposure to BPS (0.5 nM) induced a significant haplotype-dependent decrease in ABCB1 promoter activity, while chronic BPS exposure (0.3 nM) induced a significant haplotype-dependent promoter activity increase, thus dramatically impacting on P-gp levels and fetal exposure to xenobiotics coming from the maternal circulation [92]. Moreover, prenatal exposure to BPS (5 mg/kg/d) in C57BL/6 N mice was described to increase the susceptibility to high-fat-diet-induced adipogenesis in F1 male adult mice via the upregulation of PPAR-γ and its target genes [93]. Finally, the epidermal growth factor receptor (EGFR) was identified as a key mediator of phthalates effects on early placental function among a group of 39 first-trimester placental genes with altered methylation after high phthalate exposure [94].

Indeed, different EDCs types such as BPA and phthalates have specific gene targets and their actions could be influenced by fetal sex. Importantly, so-called safer alternatives to BPA as BPS could have detrimental effects on fetal programming too. These data open new perspectives into the understanding of EDCs’ influence on the prenatal development of adult health susceptibilities and pregnancy-related disorders.

## 6. EDCs and Placenta-Related Conditions

Beside impacting on epigenetic switches and fetal programming, BPA and phthalates could directly alter pregnancy physiology, undermining its success. As reported for EDC-induced epigenetic modifications, most of the mechanistic data present in the literature about endocrine disrupting chemicals and human pregnancy disorders derive from animal and in vitro data.

Starting from the very beginning of gestation, EDCs were recognized as potent perturbators of human Chorionic Gonadotropin (hCG) production and secretion [95]. hCG is a hormone specifically produced by the syncytiotrophoblast whose major functions are crucial for pregnancy establishment as it induces ovulation, maintenance of the corpus luteum and progesterone production during the first 9 weeks of gestation [95]. In vitro studies on trophoblast cell lines and human chorionic villous explants demonstrated that very low BPA concentrations increased hCG secretion and reduced extravillous trophoblast migration and invasion [96,97], early modifications typical of severe pregnancy-related syndromes such as Preeclampsia (PE).

PE is a major cause of mortality and morbidity worldwide, complicating 3 to 8% of all pregnancies and characterized by maternal hypertension and multiorgan damage. It originates during early gestation and, despite its etiopathogenesis remaining unclear, it is widely accepted that a placental/systemic vascular dysfunction characterized by an imbalance between pro- and anti-angiogenic factors such as placental growth factor (PlGF) and soluble fms-tyrosine kinase (sFlt)−1 underlie PE onset.

In a recent population-based prospective cohort study including 1233 women, a positive association was reported between increased first-trimester urine high molecular weight phthalates metabolites and a higher sFlt-1/PlGF ratio, parameter used for PE screening [98]. However, the authors did not find consistent associations among early pregnancy EDCs metabolite concentrations and maternal prenatal blood pressure, placental hemodynamic outcomes or gestational hypertensive disorders [98]. In line with these results, it was previously shown that maternal urine BPA and DEHP metabolites are associated with an increased maternal plasma sFlt-1/PlGF ratio, where BPA specifically induced sFlt-1 increase, while DEHP accounted for PlGF decrease [99].

Among the proposed mechanisms by which EDCs could lead to PE, the disruption of normal placental development plays a major role. A pregnant mouse model exposed to a BPA minimum effective dose of 4 μmol/L developed preeclampsia-like features such as hypertension, abnormal circulating and placental sFlt-1/PlGF levels and kidney damage [91]. The BPA-induced PE phenotype was accompanied by decreased trophoblast invasion, increased expression of metalloproteinases inhibitors TIMP-1/2 and DNA methylation transferase-1 (DNMT-1) and decreased expression of metalloproteinases MMP-2/9, β-catenin and WNT-2, key cell fate regulator. Of note, the BPA-related reduced expression of WNT-2 was correlated with increased DNA methylation in its promoter region [91].

Preeclampsia is often associated with Fetal Growth Restriction (FGR) defined as failure of the fetus to achieve its genetically determined growth potential [100]. DEHP exposure during pregnancy (50–200 mg/Kg) was demonstrated to induce FGR by disrupting placental thyroid hormone receptor (THR) signaling in mice [101]. The DEHP-FGR mice were also characterized by the down-regulation of THR downstream genes such as Vegf, Pgf, Igf1 and Igf2, pivotal for placental angiogenesis [101]. Other EDCs, such as fenvalerate, a widely used type II pyrethoid pesticide, were reported to induce FGR by impairing THRs pathways [102]. Specifically, maternal fenvalerate exposure down-regulated TRalpha1 and TRbeta1 placental expression and it repressed the nuclear translocation of placental TRbeta1 in mice [102]. These results are in agreement with increasing evidence demonstrating the important role of thyroid hormone receptor signaling in the preservation of placental function and fetal development [103,104].

Even BPA exposure was linked to FGR development. In a recent work, a considered safe dose of BPA (50 μg/kg BPA/day) was administered to pregnant mice during the implantation window (day 1 to 7 of gestation), thus inducing defective remodeling of maternal spiral arteries by placental trophoblast with consequent intrauterine growth restriction [105].

The deleterious EDCs effects on physiological pregnancy development were also demonstrated in in vitro human placental models and population studies, even though contrasting data are present in the literature. MEHP inhibited trophoblast invasion by activating the PPARγ pathway in human HTR-8/SVneo extravillous trophoblast cell lines, mechanisms associated with early pregnancy loss [106]. A prospective birth cohort investigation performed on 788 mother–child pairs in the third trimester in Korea concluded that BPA exposure was negatively associated with intrauterine linear growth and affected by maternal glutathione transferases polymorphisms [107], while previous studies reported no association between exposure to BPA and birth weight [108].

In conclusion, despite controversial evidence in epidemiologic, in vitro and animal studies, there is a general consensus on the harmful effects of EDC exposure during fetal life. Several molecular mechanisms have been proposed to explain the role of BPA and phthalates in the onset of severe pregnancy-related conditions such as PE and FGR, suggesting as a main outcome an aberrant placental development. Most of the differences in the literature results and interpretations are probably due to an extreme variability in EDC types and concentrations used, time of administration, for animal or in vitro models, or sampling windows for human gestation studies.

## 7. EDCs and Gestational Diabetes

It is well known that even during a healthy pregnancy, the release of numerous placental hormones promotes insulin-resistance, which is addressed through a compensatory insulin secretion by the pancreatic β-cells [109]. Gestational diabetes (GDM) occurs when pregnant women have dysfunctional β-cells, unable to balance the increased requirements of insulin [110].

Excessive weight gain during pregnancy is a risk factor for GDM. Some recent studies suggest a possible link between the gestational exposure to EDCs and weight gain in pregnancy. In a population-based prospective cohort study among 1213 pregnant women, each log unit increase in early pregnancy BPA and di-n-octylphthalate urine concentrations were associated with lower mid-to late pregnancy gestational weight gain (−132 g/log unit increase [95% CI −231, −34] and −176 g/log unit increase [95% CI −324, −29]) [111]. Differently, in the LIFECODES pregnancy cohort, 347 pregnant women were recruited, and the 1^st^-trimester urinary phthalate metabolite concentrations were related to early gestational weight gain (median time period: 7.4 gestational weeks). The association between MEP and gestational weight gain followed a U-shape with increasing gestational weight gain in the second quartile compared to the lowest one (difference 1.3 kg, 95% CI: 0.3–2.4). Differently, the dose–response association between DEHP and gestational weight gain was described by an inverse U-shape [112].

In a Chinese cohort prospective study including 620 pregnant women, plasma glucose at 2 h in the 75-g OGTT was 0.36 mmol/L lower (95% CI −0.73, 0.01) for women with urine BPA in the high versus the low tertile, and for each log unit increase, the odds of GDM was reduced by 27% (OR 0.73, 95% CI 0.56, 0.97) [113]. On the contrary, in a small case-control study, no evidence of association between BPA exposure and GDM diagnosis across increasing tertiles of BPA exposure was found [114]. No statistically significant associations were observed between first-trimester urinary BPA concentration with diagnosis of impaired glucose tolerance (IFG) or GDM even in the Maternal-Infant Research on Environmental Chemicals (MIREC) cohort study [115]. The latter study also failed to find an association between urinary concentration of phthalates metabolites and risk of IFG and GDM [115]. Contrary, in the LIFECODES pregnancy cohort, second-trimester MEP exposure was associated with increased odds of IGT (OR 7.18, 95% CI 1.97, 26.15). DEHP concentration was inversely associated with IGT (OR 0.25, 95% CI 0.08, 0.85). In both cases, the confidence intervals were very wide, suggesting low accuracy of the risk estimates [116]. In “The Infant Development and Environment Study” (TIDES), which included 705 pregnant women, the averaged first and third trimester urinary MEP concentration was associated with increased odd of GDM (OR 1.61, 95% CI 1.10, 2.36), whereas first-trimester urinary mono-(3-carboxypropyl) phthalate (MCPP) concentration was inversely associated (OR 0.64 95% CI 0.43, 0.96). Only the averaged first- and third-trimester urinary mono-n-butylphthalate (MNBP) concentration was associated with the risk of IGT (OR 1.32, 95% CI 1.00, 1.75) [117]. In a small cross-sectional study, women with the highest urinary concentrations of mono-iso-butyl phthalate (MIBP) and mono-benzyl phthalate (MBZP) had lower blood glucose levels at the time of GDM diagnosis compared to women with lower urinary concentrations of such phthalates metabolites [118].

In conclusion, the limited evidence and conflicting results do not allow a definitive conclusion. The discrepancies in the literature results are presumably due to the different time windows considered for the exposure assessment, the use of a single-spot urine sampling to assess exposure, the different criteria used to diagnose GDM, and not considering pre-gestational exposure.

## 8. Guidelines to Reduce Dietary Exposure to EDCs

Although there is an urgent need to have data on the association between the level of EDCs in reproductive age and pregnancy outcomes, it is well known that dietary exposure is relevant. There can be substantial variability in phthalate concentrations within food groups based on the region of food production, processing practices, presence and type of packaging and lipid content [119,120]. Recent reviews of the food monitoring and epidemiology data revealed that foods of animal origin are major sources of phthalates, partially because they are slightly lipophilic and can bioaccumulate in fat-containing foods [10,121]. Poultry, some dairy products (cream) and fats are routinely contaminated with higher concentrations of phthalates than other foods [121]. Detectable concentrations of phthalates and BPA are in seafood products, especially if frozen or canned [10], and consuming them ≥1–3 times/week making it a major source of BPA during pregnancy [122]. By contrast, milk, yogurt and eggs were found to contain low concentrations of phthalates as a whole [121]. Concerning vegetable foods, fruit and vegetables products in jars contain high concentrations of phthalates, while fresh fruits and vegetables, pasta, noodles, rice are associated with lower exposures [10].

### 8.1. Perfluorinated Compounds (PFCs): PFOS and PFOA

According to the EFSA Panel on Contaminants in the Food Chain, some foods (especially seafood) are an important source of exposure to PFCs. Moreover, PFOA has been used in the past in the production of non-stick coatings. Currently, Italian manufacturers of cookware coatings do not use products with PFOA. Therefore, consumers have to turn their attention to products coming from non-European countries, especially those without the CE mark [123].

### 8.2. Di(2-Ethylhexyl)-Phthalate (DEHP)

Food contact materials (film, blister packaging, screw caps, bottles, trays and transport packaging) are the main dietary sources of DEHP. However, DEHP use in Europe has dramatically dropped; for some usages, such as flooring and food contact film, European manufacturers have almost completely substituted and phased-out DEHP [123].

### 8.3. Polycyclic Aromatic Hydrocarbons (PAHs)

PAHs are a group of compounds that are formed from combustion processes, both industrial and household. Nutrition plays a key role in the prevention of exposure: PAHs form at high temperatures within overheated parts of foods, especially with some cooking methods, such as grilling or charring, smoking, or barbecuing [123].

### 8.4. BPA

As mentioned above, BPA is a chemical «building block» for the manufacture of polycarbonate plastic used in food contact materials and epoxy resins (lining protective of most cans and food recipients). Canned foods and beverages are the main source of dietary exposure for all age groups, and consumers may be exposed to either the residual monomer that migrates from cans to beverages and foods, or the products that result from polymer hydrolysis [123].

Table 2 summarizes the practical arrangements for food selection, cooking and storage elaborated by the National Institute of Health [123] and integrated with other recent epidemiological findings [122,123,124,125] to reduce dietary exposure to EDCs during reproductive age and the risk of developing pathological conditions that may compromise a future pregnancy.

## 9. Concluding Remarks

Endocrine-disrupting chemicals represent a health threat that should not be underestimated, especially when dealing with human pregnancy. Pregnant women can be easily exposed to a large number of EDCs by dietary intake. Because of their biochemical features, EDCs pass the placental barrier and reach the developing fetus, causing aberrant genetic/epigenetic regulation with sex-specific modifications and contributing to the onset of placenta- and pregnancy-related disorders. The medical-scientific community has still a lot of work to do in order to clarify the pathways involved in EDCs’ effects on pregnancy physiology. The main limitation that explains the contrasting results present in the literature is the high variability in experimental conditions and models used to investigate EDC activity in the perinatal period. Therefore, a great effort must be made by researchers in the field to set up common protocols and guidelines in order to improve data reliability.

## Figures and Tables

**Table 1:** Endocrine-disrupting chemicals (EDCs) most relevant to human health.

EDC	Metabolites	Exposure Sources
**Bisphenol** [10] Bisphenol A (BPA) Bisphenol S (BPS) Bisphenol F (BPF) Bisphenol B (BPB)	[26] BPA glucuronide (BPA-G) BPA sulfate (BPA-S)	Synthetic [10] Food packaging; Thermal receipts; Plastic dinnerware; Polycarbonate plastic; Epoxy resins; Dental sealants;
**High-Molecular-Weight Phathalate** [10] Di(2-ethylhexyl) phthalate (DEHP)	[10] Mono(2-ethyl-5-hydroxyhexyl) phthalate; mEHHP Mono(2-ethylhexyl) phthalate; mEHP Mono(2-ethyl-5-oxohexyl) phthalate; mEOHP Mono(2-ethyl-5-carboxypentyl) phthalate; mECPP	Synthetic [10] Food packaging and processing; Pharmaceutical coatings; PVC plastics; Building materials; Medical devices;
**Low-Molecular-Weight Phathalate** [10] Diethyl phthalate (DEP)	[10] Monoethyl phthalate; mEP	Synthetic [10] Fragrant PCPs:| perfumes/colognes; deodorants; soaps, shampoos lotions;
**Persistent Organic Pollutants (POPs)** Dichlorodiphenyltrichloroethane (DDT) [27] Dioxins (PCDD, PCDF) [5]	chlorodiphenyldichloroethylene (DDE)	Synthetic Pesticides; [27] Insecticide; Combustion; [5] Incineration; Waste burning; Paper bleaching;
**Polycyclic Aromatic Hydrocarbons (PAHs)** [27] polybrominated diphenyl ethers (PBDEs) polychlorinated biphenyls (PCBs) brominated flame retardants (BFRs)		Synthetic [27] Combustion processes; Building materials; Electronics furniture; Hydraulic fluids;
**Perfluorinated Alkylated Substances (PFAS)** [7] Perfluoroalkyl Polyfluoroalkyl		Synthetic [7] Personal care products: Polishes and Paints; Non-stick cookware; Fire-fighting foams;
**Phytoestrogens** [8] Isoflavonoids (Genistein, Daidzein)		Natural [8] Soy beans and other legumes

**Table 2:** Guidelines to reduce dietary exposure to EDCs.

**Food Selection**
1. Prefer fresh seasonal food, especially fish, fruits and vegetables;
2. Reduce the consumption of canned fish or frozen seafood to once a week;
3. Buy your tomato sauce and legumes in glass jars;
4. Purchase beverages in plastic or glass bottles. Prefer tap water if possible: visit the website of your municipality to learn more about its characteristics;
5. Avoid ready-made food as “heat-and-go” cups or instant soups;
6. Prefer pizza or sandwich without boxes or wrappers but displayed freshly at the counter;
7. Reduce the use of popcorn bags for microwave cooking, choosing stovetop alternatives;
8. Replace a plastic coffee maker using a French press or ceramic drip; 9. Avoid plastic tea bags and purchase tea from manufacturers who can certify that their tea bags do not contain EDCs. It is preferable to opt for loose tea;
10. Avoid the consumption of partially charred/burned foods, removing burned parts (i.e., meat or pizza).;
11. Limit smoked foods to once a month.
**Cooking**
12. Avoid heat and use only undamaged containers to heat food and beverages and only for the uses specified by the manufacturer;
13. Do not put polycarbonate plastics in the microwave. Use glass, porcelain or stainless-steel containers for hot foods and liquids in place of plastic containers;
14. Remove the fatty portion of meat before cooking: reduce barbecuing or grilling, especially those over charcoal, preferring other cooking methods;
15. Phase out the use of worn non-stick cookware, even to ensure cooking without charring/carbonization. Turn your attention to products coming from non-European countries, especially those without the CE mark;
16. When cooking, ensure a proper ventilation in the room and use an appropriate kitchen range hood.
**Storage**
17. Use dishwasher only for plastic containers suitable for high temperatures;
18. Do not reuse worn out plastic containers for food and beverages;
19. Let hot food and beverages cool before pouring in plastic containers not suitable for high temperatures;
20. Use grease-proof paper or film for food packaging (e.g., cling film) only following the manufacturer’s instructions. Read the product’s label;
21. When choosing home materials, limit the use of soft PVC containing DEHP. Prefer BPA-and phthalates-free products.
